# An Extraordinary Gobioid Fish Fossil from Southern France

**DOI:** 10.1371/journal.pone.0064117

**Published:** 2013-05-15

**Authors:** Christoph Gierl, Bettina Reichenbacher, Jean Gaudant, Dirk Erpenbeck, André Pharisat

**Affiliations:** 1 Department of Earth and Environmental Sciences, Ludwig-Maximilians University, Munich, Germany; 2 Muséum National d’Histoire Naturelle, Paris, France; 3 Museum G CUVIER, Montbéliard, France; Team ‘Evo-Devo of Vertebrate Dentition’, France

## Abstract

**Background:**

The classification of gobioid fishes is still under discussion. Several lineages, including the Eleotridae and Butidae, remain difficult to characterize because synapomorphies are rare (Eleotridae) or have not yet been determined (Butidae). Moreover, the fossil record of these groups is scarce.

**Results:**

Exceptionally well-preserved fish fossils with otoliths *in situ* from uppermost Oligocene sediments (≈23–24 Mio. y. ago) in Southern France provide the most in-depth description of a fossil gobioid to date. The species was initially described as *Cottus aries* Agassiz, then transferred to †*Lepidocottus* Sauvage, and subsequently assigned to *Gobius*. Based on a comparative analysis of meristic, osteological and otolith data, this species most likely is a member of the family Butidae. This discovery is important because it represents the first record of a fossil butid fish based on articulated skeletons from Europe.

**Significance:**

The Butidae and Eleotridae are currently distributed in W-Africa, Madagascar, Asia and Australia, but they do not appear in Europe and also not in the Mediterranean Sea. The new results indicate that several species of the Butidae thrived in Europe during the Oligocene and Early Miocene. Similar to the recent Butidae and Eleotridae, these fishes were adapted to a wide range of salinities and thrived in freshwater, brackish and marginal marine habitats. The fossil Butidae disappeared from Europe and the Mediterranean and Paratethys areas during the Early Miocene, due probably to their lack of competitiveness compared to other Gobioidei that radiated during this period of time. In addition, this study documents the great value of otoliths for gobioid systematics.

## Introduction

The Gobioidei represents one of the most species-rich vertebrate suborders, with approximately 2,000 extant species (belonging to >270 genera) thriving in marine, estuarine and freshwater habitats [1], [2]. Their classification was initially based on typical complements of morphological characters (e.g. [2]–[11]), and, more recently, largely confirmed by studies using molecular data ([12]–[16], for a review see [17]). Six family-based clades are currently recognized, i.e. the Rhyacichthyidae, Odontobutidae, Eleotridae, Gobiidae, Gobionellidae and Butidae [13]. Moreover, the family state of the Milyeringidae was supported by Chakrabarty [18], and the new family Thalasseleotrididae was introduced by Gill and Mooi [19]. However, the large number of species and generally small size of individuals, a tendency towards evolution by reduction, and a wide range of specializations make several aspects of the Gobioidei systematics still difficult to understand. One example is the classification of the Butidae and Eleotridae. Thacker [13] used molecular data to elevate the two previous subfamilies of the Eleotridae (Butinae, Eleotrinae, see [1], [7], [20]) to the rank of family, Butidae and Eleotridae (see also [21]), but synapomorphies based on morphological traits have not yet been determined for the Butidae. Although various details of Thacker’s classification [13] continue to be controversial [2], [17], [22], [23], the eleotrids and butids are now unanimously considered as belonging to two different families (e.g. [24], [25]).

The fossil record represents a very important source of direct information for the understanding of the evolution and phylogeny of organisms. The fossil record of modern bony fishes (teleosts) is based on articulated skeletons and isolated otoliths; however, skeletons and otoliths are typically found separated, and most fossil teleosts are based exclusively on articulated skeletons or isolated otoliths. This is also true of the fossil gobioids, of which a few articulated skeletons and a relatively large number of isolated otoliths are known. The oldest articulated gobioid skeleton was discovered in the Middle Eocene (≈44 Mio. y. ago) of Catalonia (Spain) [26], and the oldest gobioid otoliths come from the Lower Eocene (≈52 Mio. y. ago) of India [27]. However, determining the generic and sometimes even the familial affinities of fossil gobioid skeletons and otoliths is very difficult. Skeletons may exhibit synapomorphies of more than one extant family [28] or the synapomorphies are not preserved [26]. Synapomorphies of gobioid otoliths have not been identified to date and thus otoliths are usually identified by comparison with the otoliths of extant gobioids (e.g. [29]–[32]). In addition, most previous studies on fossil gobioids have focused on past diversity and zoogeography; this explains why character analyses or identification of synapomophies were usually not provided. As a result, many fossil gobioids, skeleton-based species as well as otolith-based taxa, have been assigned to the genus *Gobius* Linnaeus *sensu lato*, but may in fact belong to other gobioid genera and/or families (e.g. *Gobius brevis* (Agassiz), see [33]).

This study is based on a critical re-evaluation of the extinct genus †*Lepidocottus* Sauvage and its type species †*L. aries* (Agassiz) from the Upper Oligocene of southern France. Some of the specimens studied here are exceptional in that they display both the cranium and otoliths *in situ*. †*Lepidocottus aries* was originally assigned to the Cottidae [34], [35], but later transferred to the gobiid genus *Gobius* Linnaeus [36], [37]. Our results show that †*Lepidocottus* is a member of the Gobioidei, but not a gobiid. Rather, it represents the first fossil record of a close relative of the extant Butidae from Europe that is based on articulated skeletons. Since the fossils included in our study are exceptionally well preserved, with several bones of the skull and fins in three-dimensional preservation, and stomach content still in place, we provide the most in-depth description of a fossil gobioid to date.

### Geological Setting

**Figure 1:**
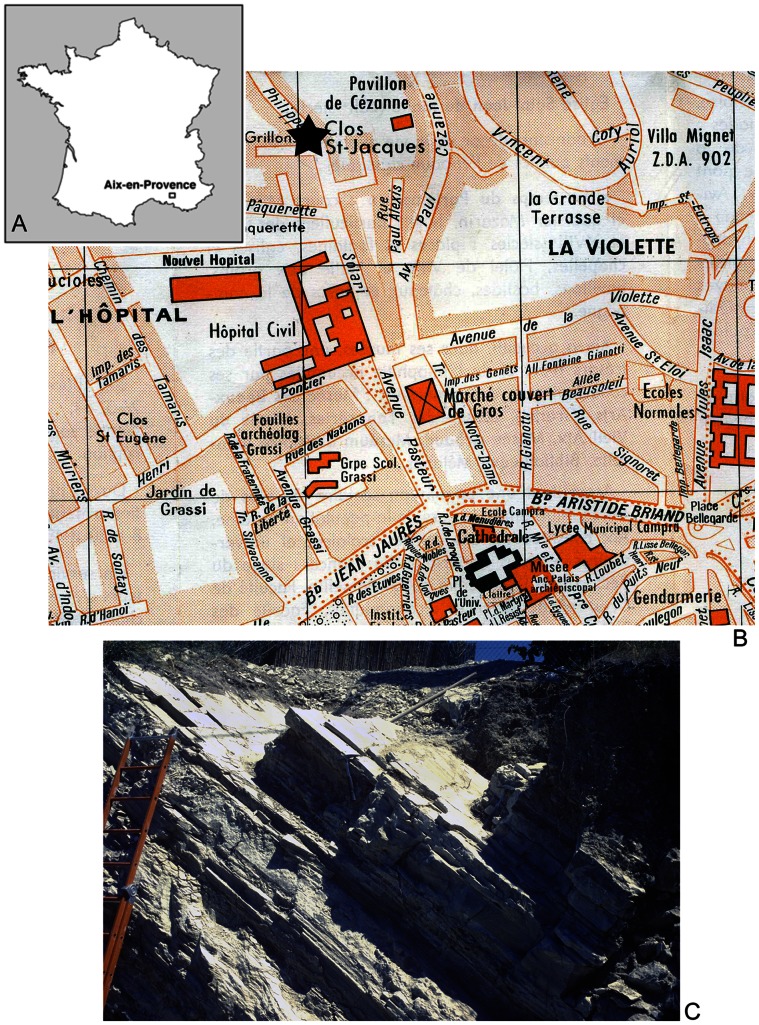
Geographic overview and sediments of the studied site. **A.** Location of Aix-en-Provence in southern France. **B.** Position of the fish fossil-bearing outcrop (indicated by star) in Aix-en-Provence. **C.** Lithofacies of the sediments (hammer for scale) at the studied outcrop.

During the Oligocene, several continental basins developed in the Provence and Languedoc areas in southern France, among them the basin of Aix-en-Provence or Aix-Basin [38]–[40] (Fig. 1). Towards the end of the Oligocene and earliest Miocene, this basin was irregularly connected to the Mediterranean Sea, of which the shore-line was then located some 20 km to the South, approximately where the city of Marseilles lies today. The sedimentary filling of the Aix-Basin is about 150 m thick and termed Aix-en-Provence Formation [41]; its description is mainly based on the lithology of the 80 m deep drilling at Puy-du-Roy (about 3 km N-NW of the city center of Aix-en-Provence) and was complemented by outcrop observations along the road from Aix-en-Provence to Avignon (avenue Maréchal de Lattre de Tassigny). According to Nury [41], the Aix-en-Provence Formation can be subdivided into seven members. The lowermost member consists of marls alternating with beds of conglomerates, it is termed Marnes et Conglomérats de Sainte Anne. At its top appears a lignitic fossiliferous intercalation that has yielded the rodent *Rhodanomys schlosseri* Deperet & Douxami associated with *Wenzia ramondi* (Brongniart) and other gastropods. This fossil assemblage was considered as typical for the Late Oligocene [41], however, *Rhodanomys schlosseri* may also indicate an Early Miocene age [42]. The second member is named Calcaires et Marnes des stations d’essence and contains gastropods (*Potamides lamarckii* Brongniart, planorbidids), bivalves (*Pisidium* sp.) and fishes (*Dapalis* sp.). The gobioid skeletons and otoliths described in this study come from this member. Above follow the Calcaire et Marnes à gypse d’Aix, which have long been mined in underground galleries and yielded the famous fish fauna described by de Blainville [43] and Agassiz [34]. The remaining members of the Aix-en-Provence Formation consist of limestones, marls and sands (see [41], [44]).

## Materials and Methods

Specimens were collected in 1974 by one of us (AP) in the city of Aix-en-Provence, at the corner of Avenue Philippe Solari and Chemin du Pin, during the construction of a house [45] (Figs. 1B–C). The lithofacies of the laminated marly sediments indicates that they belong to the Aix-en-Provence Formation and most probably to its second member, i.e. the Calcaires et Marnes des stations d’essence. A more precise correlation is not possible because, contrary to the feebly inclined strata of the Aix-en-Provence Formation along the road to Avignon, the strata of Chemin du Pin show a rather steep eastward dip (about 40°) (Fig. 1C).

The material is comprised of nine articulated skeletons today deposited in the Palaeontological collection of the Museum G. Cuvier; Montbéliard (France), under accession numbers MC-P-2011-01-TF1 to -TF9. Four of the skeletons possess saccular otoliths *in situ*. In addition, three specimens were available for comparison from the collections of the Hessian State Museum in Darmstadt (HLMD SMFF-356, determined as *Gobius aries* (Agassiz); HLMD 1910-V-2543, determined as *Ophidion barbatum* Linnaeus) and the Museum for Natural History in Vienna (NMW 1910-V-12, determined as *Gobius aries*).

Osteological, meristic and morphometric characters of the skeletons and otolith characters were studied under a stereomicroscope equipped with a digital camera. Measurements were taken with a calliper to the nearest 0.1 mm. The D1 pterygiophore formula (e.g. 4(22110)) follows Birdsong et al. [5]; the first number indicates the position of the interneural space with the first pterygiophore (e.g. behind vertebra 4), each figure within the brackets represents an interneural space, starting with the one into which the first pterygiophore is inserted (e.g. behind vertebra 4), and the number indicates the number of pterygiophores inserting at that position (e.g., two pterygiophores behind vertebrae 4 and 5, respectively; one pterygiophore behind vertebrae 6 and 7, respectively; no pterygiophore behind vertebra 8).

**Figure 2:**
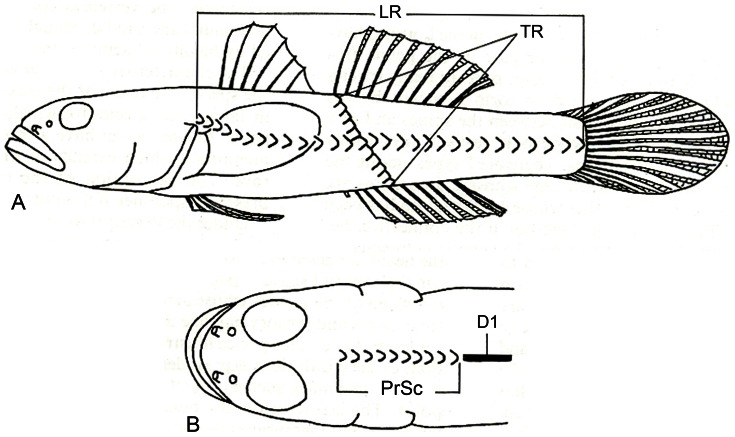
Method for counting scales (from [46]). **A.** Counting of the numbers of scales in the longitudinal row (LR) and transverse row (TR). **B.** Counting of the predorsal scales (PrSc) in the dorsal midline in front of the dorsal fin.

The counts of principal caudal fin rays refer to the number of segmented and branched rays. The counts of the predorsal scales and the number of scales in the longitudinal and transverse rows follow Masuda et al. [46]. The number of longitudinal scales equals the number of scales in the lateral line series that has frequently been used in other studies; the number of predorsal scales is counted at the midline of the fish, from the insertion of the first dorsal fin toward the head (Fig. 2).

Information on the skeletons of extant gobioids for comparison with our fossils was gathered from the primary literature, mainly from the studies by Regan [47], Hoese [6], Akihito et al. [48], Birdsong et al. [5], Harrison and Miller [49], Hoese and Gill [7], Johnson and Brothers [50], Pezold [10], Winterbottom [51], Miller [9], Akihito et al. [12], Larson and Murdy [52], Harrison et al. [53], Kindermann et al. [54], Froese and Pauly [55], and Gill and Mooi [19]. The information used in the comparison of the otoliths was obtained from specimens of extant eleotrids and butids kept in the collections of the Institut Royal des Sciences Naturelles de Belgique and of Dr. W. Schwarzhans (Hamburg).

Institutional abbreviations used: HLMD, Hessian State Museum in Darmstadt, Germany; IRSNB, Institut Royal des Sciences Naturelles de Belgique, Brussels, Belgium; MC, Museum G. Cuvier, Montbéliard, France; NMW, Museum of Natural History of Vienna, Austria; USNM, National Museum of Natural History, Smithsonian Institution, Washington, D.C., U.S.A.; WAM, Western Australian Museum, Perth, Western Australia; ZMH, Zoological Museum Hamburg, Germany; ZMUC, Zoological Museum, Copenhagen, Denmark.

No permits were required for the described study, which complied with all relevant regulations.

## Results

### Preliminary Remark

The studied specimens are determined as †*Lepidocottus aries* (Agassiz), because they largely correspond to the original description of this species by Agassiz [34] (p. 12, 186–187) and were found at the type locality (Aix-en-Provence). In addition, our specimens correspond well to specimens determined as *Gobius aries* from previous collections at Aix-en-Provence (NMW 1910-V12, HLMD SMFF-356).

Agassiz has indicated that his new species *Cottus aries* is figured on Plate 18; however, this plate was never printed and thus no figure of the holotype exists (see also [35]: 635). Our efforts to find the holotype in the Natural Museum of History, Paris yielded no success, and thus the holotype has to be considered as lost. Sauvage [35] considered, like Agassiz, *Cottus aries* as a member of the Cottidae, but recognized that it is different from the extant *Cottus* Linnaeus and therefore introduced the new genus name †*Lepidocottus* for it. Sauvage [35] provided a description and also a figure of †*L. aries* (Agassiz) based on two newly collected specimens from Aix that he had received from a private collector, but also these specimens are apparently lost.

### Systematic Palaeontology

The classification follows Nelson [1]. For a newly proposed classification, see also Wiley and Johnson [56].

Order Perciformes Bleeker, 1859

Suborder Gobioidei Agassiz, 1835

Family Butidae Bleeker, 1874 (originally as Butii)

Genus †*Lepidocottus* Sauvage, 1875

**†** ***Lepidocottus aries*** **(Agassiz)**

**Figure 3:**
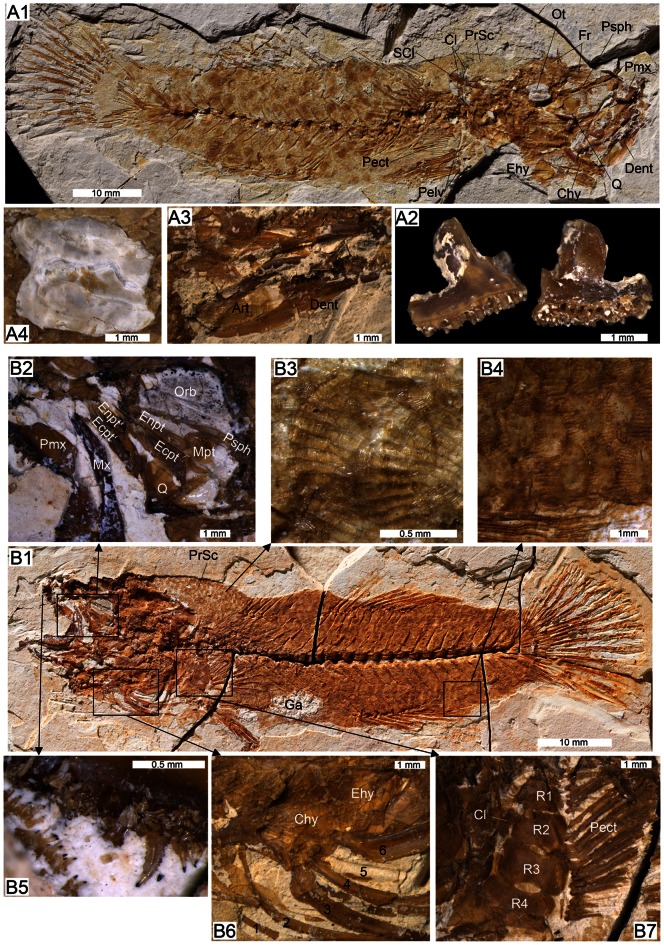
Osteology, scales and otolith of †*Lepidocottus aries* (Agassiz). A: Specimen MC-P-2011-01-TF1. B: Specimen MC-P-2011-01-TF2. **A1.** General overview. Head displays right premaxilla (Pmx) and frontal (Fr) in lateral view, and several bones from the left head side in medial view (dentary (Dent), quadrate (Q), anterior ceratohyal (Chy), posterior ceratohyal (epihyal, Ehy)). The elongate parasphenoid (Psph) is also visible. The girdle exposes both pelvic fins (Pelv), the left supracleithrum (SCl), and imprints of the left pectoralis (Pect) and the uppermost part of the left cleithrum (Cl). The predorsal scales (PrSc) are well preserved. **A2.** Close-up of right premaxilla (isolated from skeleton) showing alveoles for the teeth and a complete processus articularis. Lateral (left) and medial views (right). The processus ascendens is broken and not preserved. **A3.** Close-up of left dentary with angulo-articular (Art), medial view. **A4.** Close-up of left saccular otolith, inner face. **B1.** General overview. The head displays several bones from the right sides of the head and the girdle in medial view (see B2–B7 for details). The predorsal scales are well visible. The stomach and gut region bears numerous gastropod shell fragments (Ga). **B2.** Orbital and ethmoidal region (right side, medial view), showing the orbit (Orb) with the supra- and postorbital crests of the frontal, the almost articulated area of the quadrate, the ectopterygoid (Ecpt, Ecpt’) and entopterygoid (Enpt, Enpt’) from both parts of the skull, the right metapterygoid (Mpt, fragment), and the posterior processus of the right premaxilla. **B3.** Close-up of predorsal cycloid scale with well-developed circuli and radii. **B4.** Close-up of ctenoid body scales with radii and ctenii. **B5.** Close-up of premaxillary teeth. **B6.** Close-up of right anterior ceratohyal, branchiostegal rays (1–6) and part of the subopercle (Sop). Three branchiostegals from left body side are also visible. **B7.** Close-up of right pectoral fin with well-developed radials (R1–4) and remains of cleithrum.

**Figure 4:**
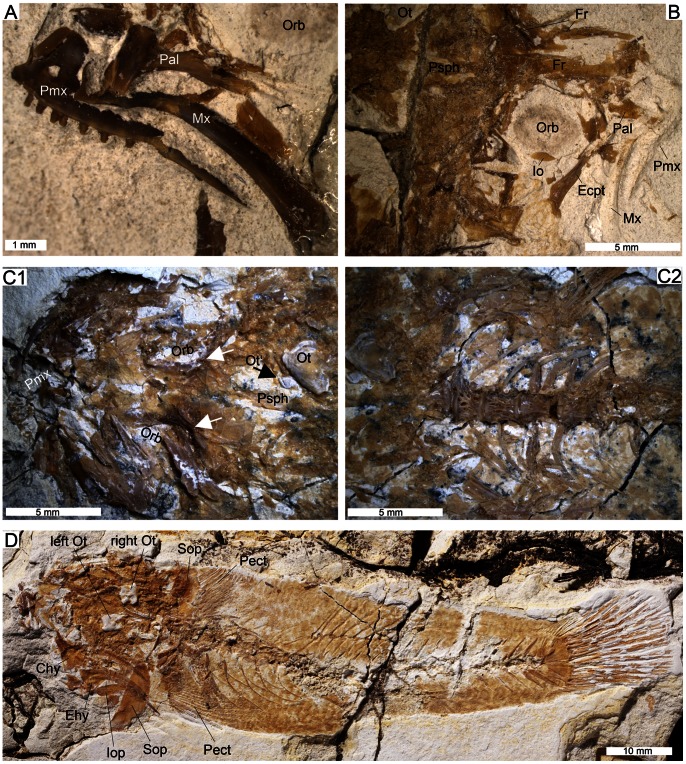
Details of the osteology of †*Lepidocottus aries* (Agassiz). **A.** Specimen MC-P-2011-01-TF6, left head side in lateral view showing premaxilla (Pmx), maxilla (Mx), L-shaped palatine (Pal), and orbit (Orb). **B.** Head of Specimen MC-P-2011-01-TF4 in dorso-right-lateral view showing orbit (Orb) with infraorbital (Io), moderately preserved frontal bones (Fr), mesethmoid, remains of L-shaped palatine with attached ectopterygoid (Ecpt), imprints of maxilla and premaxilla. This specimen bears a right saccular otolith preserved *in situ* (not shown). **C1.** Specimen MC-P-2011-01-TF5, dorsal view of the head with orbits (Orb) and frontal bones. Prominent crests (white arrows) of frontals represent supra- and postorbital sensory canals. Left saccular otolith (Ot) and right utricular otolith (Ot’) are preserved *in situ*. The parasphenoid (Psph) is also visible. **C2.** Specimen MC-P-2011-01-TF5, anterior part of vertebral column, showing details of centra and ribs. Note that neural spines are not preserved (broken) at vertebrae 1 to 3. **D.** Specimen MC-P-2011-01-TF3, general overview. The head is preserved in dorsal view and displays the otoliths of both head sides and the subopercle (Sop), anterior ceratohyal (Chy), posterior ceratohyal (epihyal, Ehy), branchiostegal rays, spiny gill rakers and interopercle (Iop) of the left side (medial view). The girdle exposes both pectoral fins (Pect). Ribs and epipleurals, the second dorsal fin and the caudal endoskeleton and fin are well preserved. The number of branched caudal fin rays is 7/6.

**Figure 5:**
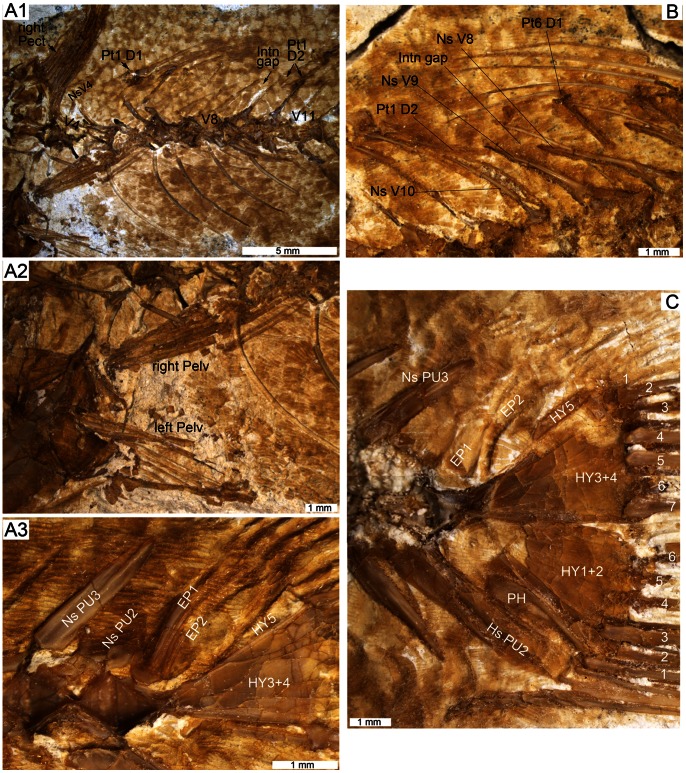
Details of the postcranial skeleton of †*Lepidocottus aries* (Agassiz). **A1.** Specimen MC-P-2011-01-TF8, right pectoral fin (Pect) and anterior portion of the vertebral column. The first pterygiophore of the first dorsal fin (Pt1 D1) inserts behind the neural spine (Ns) of vertebra 4 (V4), and the first pterygiophore of the second dorsal fin (Pt1 D2) inserts behind the neural spine of vertebra 9 (V9). The interneural gap (Intn gap) lies between vertebrae 8 and 9. **A2.** Close-up of the putative separated pelvic fins (Pelv). **A3.** Close-up of the dorsal part of the caudal endoskeleton, showing the neural spines of the pen- and antepenultimate vertebrae (PU2, PU3; note the short Ns of PU2), the two epurals (EP1, EP2) and the dorsal hypural plates (HY3+4, HY5). **B.** Specimen MC-P-2011-01-TF7, anterior part of the vertebral column, showing the spines of the first dorsal fin, the prominent pterygiophore supporting the last spine (Pt6 D1), the interneural gap (Intn gap) between the neural spines of vertebrae 8 and 9 (Ns V8, Ns V9), and the first pterygiophore of the second dorsal fin (Pt1 D2). **C.** Close-up of caudal fin of specimen MC-P-2011-01-TF3 (see Fig. 4D), showing the expanded neural and haemal spines (Hs) of the antepenultimate vertebra (PU3), two epurals (EP1, EP2), two large hypural plates (HY1+2, HY3+4) and a small one (HY5), the parhypural (PH) and the branched rays (7/6). Note that numbering of epurals indicates position and does not imply homology.

**Figure 6:**
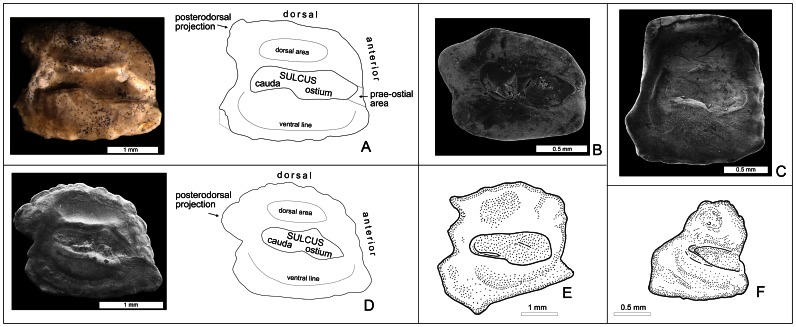
Otoliths of: †*Lepidocottus aries* (Agassiz) (A); extant Butidae (B), Eleotridae (C), Gobiidae (D) and Odontobutidae (E–F). **A.** *In situ*-preserved otolith of †*L. aries* [ = left saccular otolith, isolated from specimen MC-P-2011-01-TF5, see Fig. 4C1]; stereoscope photo and schematic drawing with terminology of characters. **B.** Otolith of the butid *Kribia kribensis* (Boulenger), West Africa (Cameron), IRSNB. **C.** Otolith of the eleotrid *Mogurnda mogurnda* (Richardson), Oceania, IRSNB. **D.** Otolith of the gobiid *Gobius niger* Linnaeus [ = mirrored right saccular otolith]; SEM photo and schematic drawing with terminology of characters, coll. W. Schwarzhans. **E.** Otolith of the odontobutid *Odontobutis obscura* (Temminck & Schlegel), Myanmar, IRSNB (drawing by D. Nolf). **F.** Otolith of the odontobutid *Micropercops swinhonis* (Günther), China, Bejing, Huwairiu county, USNM 336 833 (drawing by D. Nolf).

Figs. 3, 4, 5, 6A, Table 1

**Table 1:** Morphometric characters (in mm) and counts of meristic characters of †*Lepidocottus aries* (Agassiz).

	MC-P-2011-01-TF1 (Fig. 2A)	MC-P-2011-01-TF2 (Fig. 2B)	MC-P-2011-01-TF3 (Fig. 3D)	MC-P-2011-01-TF6
**Total length**	90	84	96	ca. 83
**Standard length**	71	69	81	ca. 75
**Maximum height of body**	17.5 (25%)	17 (25%)	23 (28%)	17 (23%)
**Number of vertebrae (precaudal+caudal)**	25 (10/11+14/15)	25 (10+15)	25	26
**Number of spines in D1**	6	6	–	
**Total number of rays in D2**	11(?12)	10	>7	11
**Total number of rays in anal fin**	10	9	–	10
**Number of rays in pectoral fin**	>9	14	14–16	>10
**Number of rays in pelvic fin**	6	6	6	5
**Number of branched principal caudal rays**	13	13	13	
**Head length**	25 (35%)	25 (36%)	25 (31%)	Ca. 17 (<23%)
**Distance snout to D1**	32 (45%)	30 (43%)	27 (33%)	–
**Distance snout to D2**	43 (61%)	41 (59%)	45 (56%)	Ca. 37 (<49%)
**Distance snout to anal fin**	47 (66%)	44 (64%)	–	Ca. 38 (<51%)
**Distance snout to pectoral fins**	27 (38%)	24 (35%)	26 (32%)	Ca. 18 (<24%)
**Distance snout to pelvic fins**	28.5 (40%)	26 (38%)	27 (33%)	Ca. 18 (<24%)
**Max. length of ray in D1**	6.5 (9%)	6 (9%)	–	
**Max. length of ray in D2**	12 (17%)	9 (13%)	>6 (>7%)	9 (12%)
**Max. length of ray in anal fin**	11.5 (16%)	9 (13%)	>9 (>11%)	
**Max. length of ray in pectoral fin**	16.5 (23%)	14 (20%)	13 (16%)	
**Max. length of ray in pelvic fin**	15 (21%)	10 (14%)	11 (14%)	
**Basal length of D1**	8 (11%)	7 (10%)	–	
**Basal length of D2**	12 (17%)	10 (14%)	12 (15%)	
**Basal length of anal fin**	12 (17%)	9 (13%)	–	
**Length of caudal peduncle**	17 (24%)	18 (26%)	18 (22%)	18 (24%)
**Min. height of caudal peduncle**	11 (15%)	10 (14%)	11 (14%)	10 (13%)
**Max. length of ray in caudal fin**	20 (28%)	18 (26%)	16 (20%)	

Values in brackets are morphometric values given in percentage of the standard length. D1 = first dorsal fin, D2 = second dorsal fin, min. = minimum, max. = maximum.

1833–43 *Cottus aries* sp. nov. *–* Agassiz, Recherches sur les poissons fossiles, Vol. IV, p. 186–187.

1875 *Lepidocottus aries* (Agassiz). – Sauvage, Notes sur les Poissons fossiles, p. 635–637, Pl. 23: Fig. 1.

1975 *Gobius aries* (Agassiz). – Gaudant, Intérêt paléoécologique de la découverte de *Gobius aries*, p. 112, Pl. 1.

1978 *Gobius aries* (Agassiz). – Gaudant, Sur les conditions de gisement de l’ichthyofaune oligocène d’Aix-en-Provence, Table 1.

1981 *Gobius aries* (Agassiz). – Gaudant, Mise au point sur l’ichthyofaune oligocène des anciennes plâtrières d’Aix-en-Provence (Bouches-du-Rhône), p. 1111.

#### Material

Nine articulated skeletons, MC-P-2011-01-TF1 to -TF9.

#### Provenance

Corner of Avenue Philippe Solari and Chemin du Pin, city centre Aix-en-Provence (Bouches-du-Rhône, France).

#### Formation

Aix-en-Provence Formation, member Calcaires et Marnes des stations d’essence.

#### Age

Latest Oligocene.

#### Geographical and stratigraphical range

†*Lepidocottus aries* is additionally known from Oligocene strata near Martigues, Bouches-du-Rhône, France [57].

#### General description

The size ranges between 70 and 96 mm total length and 51 to 81 mm standard length (SL). The body is subcylindric, with a relatively long caudal peduncle (22–26% of SL, Table 1); body depth is about one-quarter (23–28%) of SL (Table 1, Figs. 3, 4). The body is covered with ctenoid scales (Fig. 3B4), but cycloid scales occur in the predorsal region (Fig. 3B3). The lateral line is absent.

The head is large and robust; its length is included about three times in the standard length (Table 1). The orbit is of medium size, subdorsal, and slightly elliptic; the eye diameter is approximately one sixth of the head length (Fig. 3B1). The mouth is terminal, the gape oblique, with the posterior end slightly in front of the orbit (Figs. 3B1–B2).

#### Neurocranium

The ethmoid region is short and bears a short mesethmoid; other ethmoid bones and the nasal bone are not recognizable. The vomer is short and rounded and apparently bears no teeth. The frontal bones display an elongate supraorbital and a widened postorbital section and show prominent crests of the supra- and postorbital sensory canals (Figs. 4B–C1). A small infraorbital (io2 or io3) is present (Fig. 4B). In the otic region appear round epiotic bones, in four specimens with otoliths *in situ* (Figs. 3A1, 4C–D). Parietal bones are absent. The parasphenoid is visible in the lower third of the orbit (Fig. 3B2).

#### Otoliths

The shape of the saccular otolith (termed otolith in the following) is rectangular, the lateral (outer) side slightly convex and the medial (inner) side almost flat. The dorsal otolith margin displays a fine crenulation and a prominent posterodorsal projection. The posterior otolith margin is first concave (below the posterodorsal projection), then it runs slightly oblique in posterior-ventral direction and meets the ventral margin with an angle of about 70–80°. The ventral otolith margin is crenulated and faintly bent; it bears a small praeventral projection. The anterior otolith margin is slightly concave; its junction with the dorsal margin is angular or faintly rounded.

The medial side of the otolith presents a sulcus that shows the “shoe-sole-like” shape that is present in most gobioid otoliths (Fig. 3A4). The sulcus covers approximately 85% of the median otolith length and is anteriorly extended, i.e. positioned closer to the anterior than to the posterior otolith margin. A distinct, but thin line borders the tip of the ostium; a small prae-ostial area is visible between the ostium tip and the anterior otolith margin. A slightly thickened crista superior is present above the middle and posterior part of the sulcus, while a thin crista inferior appears along the entire sulcus. Above the sulcus appears the elongate, moderately incised dorsal area. The ventral line is running along the ventral rim of the otolith and ascending posteriorly.

Note that the otolith preserved *in situ* in specimen MC-P-2011-01-TF1 (Fig. 3A4) displays an ostium that seems to be widely opened to the anterior margin, but this is an artifact produced by the poor preservation of this otolith; the thin suture bordering the ostium tip is destroyed due to corrosion.

#### Branchiocranium

The general structure of the upper jaw is well exposed in several specimens. The premaxilla is bent in the anterior section and bears a prominent processus articularis (Figs. 3A1–2, 4A) and an almost rectangular-shaped, large posterior processus (Fig. 3B2); the processus ascendens is longer than the processus articularis (visible in specimen NMW 1910-V-12). The oral edge of the premaxilla has three rows of irregularly arranged alveoles and conical teeth of different sizes, i.e. small (0.08–0.17 mm), medium-sized (0.25 mm) or rather large (0.4 mm) (Fig. 3B5); the largest teeth insert mostly along the outer margin of the premaxilla. The maxilla is bent anteriorly and slightly expanded posteriorly, with a prominent articular head (Fig. 4A); it is toothless.

The lower jaw is robust, but not preserved completely. The dentary is associated with a wedge-shaped angulo-articular (Fig. 3A3), the oral edge of the dentary displays two or three rows of irregularly arranged alveoles and conical teeth of different sizes; the largest teeth reach 0.55 mm (the total length of this specimen is 90 mm).

The palatine is elongate, robust, and L-shaped, i.e. it has a prominent anterior process (Fig. 4A). The quadrate is three-dimensionally preserved in several of the specimens. It is triangular, with a well-developed posterior process and a large articular head. Posteriorly, the quadrate is articulated with the symplectic that is rod-shaped anteriorly and V-shaped posteriorly (Fig. 3A1), anteriorly it is associated with the elongate ectopterygoid and entopterygoid (Fig. 3B2). A gap between the symplectic and the preopercle is visible in the comparative material (NMW 1910-V-12, HLMD 1910-V-2543).

The hyomandibula is large, but usually damaged. The further hyoid region includes a large ceratohyal, which can be subdivided into an anterior ceratohyal and a triangular-shaped posterior ceratohyal ( = epihyal). Two narrow branchiostegal rays (rays 1–2) and four robust branchiostegal rays (rays 3–6) articulate with the anterior ceratohyal, between rays 1–2 and rays 3–6 is a distinct gap (Fig. 3B6).

The opercular bones are large, but mostly not well preserved. The subopercle is semi-circular (Fig. 4D). The crescent-shaped preopercle (best visible in specimen NMW 1910-V-12) has a lower and upper arm of almost equal length, and the opercle is rounded-triangular with the tip pointing to the ventral margin, a thickening is present at its anterior and posterior margins (visible in specimen HLMD 1910-V-2543).

Many conical pharyngeal teeth ranging in size between 0.05 and 0.35 mm are present in the branchial region of most specimens. Slender ceratobranchials and numerous, very small, spiny gill rakers are also visible (Fig. 4D).

#### Vertebral column

It comprises 25–26 (10+15–16) vertebrae (v), which have a delicate net-like structure (Fig. 4C2). The anteriormost vertebral centra (v1 to v4/v5) are less elongate and less constricted in the middle than the subsequent ones.

The neural spines of v1 to v3 are expanded and triangular. The other neural spines are of almost equal length and elongate (Figs. 3A1, B1), with the exception of the short neural spine of the second preural centrum (PU2; Fig. 5A3). In addition, the neural spines of PU3–5 are more oblique than the preceding ones; they appear at the posterior end of the respective centrum.

The haemal spines are usually as long as the neural spines, with the exception of the haemal spine of the first caudal vertebra (v11), which is slightly shortened (Fig. 5A1), and the haemal spine of PU2, which is enlarged (Fig. 5C).

All abdominal vertebrae have long and prominent parapophyses, in some specimens preserved in connection with the ribs. Seven to eight rib pairs are recognizable, the last two rib pairs are slightly shorter than the preceding ones (Fig. 5A1). The ribs are long, have a strong and thickened proximal portion, and a pointed distal tip; long epipleurals are also present (Fig. 4D). There are no supraneurals.

#### Pectoral girdle

The cleithrum is long, slender and reveals a broad to triangular widening in its ventral portion (visible in specimen NMW 1910-V-12). The supracleithrum is rather robust (Fig. 3A1). The endoskeleton of the pectoral fin consists of four well-developed hour-glass radials (R) with ovate-shaped gapes in between (Fig. 3B7). A scapula and coracoid are not recognizable.

#### Paired fins

The pectoral fins, which are inserted in the lower third of the flank, comprise 14–16 rays. The pelvic fins are inserted just under or slightly behind the pectoral fins and are probably separated (Fig. 5A2). Each pelvic fin includes one spine (visible in specimen NMW 1910-V-12) and five rays. The endoskeleton of the pelvic fin consists of an elongate, slightly triangular basipterygium (Fig. 5A2).

#### Dorsal fins

The first dorsal fin (D1) has six unbranched and unsegmented, medially paired rays, increasing in length from ray 1 to 3 and then slightly descending; the last ray follows with a short gap the preceding ones. Every ray is supported by an elongate pterygiophore, distally ending in a slightly concave depression (for the articulation of the ray) (Fig. 5B). The first pterygiophore inserts behind the neural spine of vertebra 4 and the last pterygiophore is situated behind the neural spine of vertebra 7; the D1 pterygiophore formula is 4(22110) (Figs. 5A1, B).

The second dorsal fin (D2) is inserted slightly in front of the insertion of the anal fin. The first ray may perhaps be a spine, and then follow 10 segmented and branched rays (Fig. 3B1). The first ray (or spine) is supported by two pterygiophores, which both insert in the interneural space behind the vertebra 9 (Figs. 5A1, B). Every pterygiophore supporting the rays is associated to a neural spine.

#### Anal fin

The anal fin consists of a very small spine and 8 to 9 rays. The spine and the first ray are supported by a single pterygiophore. The first three or four pterygiophores insert in front of the haemal spine of the second caudal vertebra (v12), they are slightly longer than the following ones (Fig. 3B1).

#### Caudal fin

The caudal endoskeleton bears two large, triangular hypural plates (HY1+2, HY3+4) and an additional small hypural plate in the dorsal part (HY5), which is separated by a thin suture from HY3+4 (Fig. 5C). A short and slender parhypural is present and is closely associated with HY1+2; its proximal region is reduced and does not reach the terminal centrum (Fig. 5C).

Two epurals (EP) are present (Figs. 5A3, C). The anterior one is characterized by a longitudinal median rib and pointed proximally; its proximal end is close to the terminal centrum. The posterior epural is slightly shorter than the anterior epural and also shows a longitudinal rib.

The number of principal caudal fin rays that are segmented and branched is 13. The caudal fin formula is 7/6 (Fig. 5C). In addition to the segmented and branched caudal fin rays, three long and ten short unbranched rays are present dorsally, and six rather long and an undetermined number of short rays appear ventrally (Fig. 5C).

The uppermost segmented and branched caudal fin ray is supported by HY5, the next six rays are supported by HY3+4, the next five rays are supported by HY1+2, and the parhypural supports the lowermost segmented and branched ray (Fig. 5C). In addition, the epurals and the widened neural spine of PU3 contribute to the caudal endoskeleton by supporting the dorsal unbranched rays (Fig. 5C). The expanded haemal spine of PU2 supports the first (longest) ventral unbranched ray (Fig. 5C).

#### Body scales

Ctenoid scales are present all over the body except in front of the first dorsal fin. They do not differ much in size and shape in the dorsal and ventral body parts (Figs. 3A1, B1) and display regularly arranged radii and tiny ctenii (Fig. 3B4). Specimen MC-P-2011-01-TF2 shows well preserved rows of scales with an average width of 0.9 mm for the not-imbricated part of the scale (Fig. 3B4); thus, the number of scales along the lateral series ( = longitudinal scale row, see Fig. 2) can be estimated as being about 50.

#### Predorsal scales

Cycloid scales are present in the predorsal region (Figs. 3A1, B1). They show distinct radii, numerous fine circuli, and are thinner and relatively higher than the ctenoid scales. In specimen MC-P-2011-01-TF2, the width of the not-imbricated part of a predorsal scale is about 0.5 mm and the predorsal segment that is covered with this type of scales is about 12.8 mm. Therefore the number of predorsal scales can be calculated to have been about 25.

#### Stomach content

Specimen MC-P-2011-01-TF2 displays well-preserved content of the stomach and gut (Fig. 3B1), which consists of densely packed gastropod shells.

## Discussion

### Comments on Previous Studies

It should be mentioned that there are some differences between our results and the descriptions given by Agassiz [34] and Sauvage [35]. Both observed a lower number of rays in the second dorsal fin and pelvic fin (nine and four, vs. eleven and six in our specimens). Moreover, Sauvage [35] reported spines both on the preopercle and opercle (not visible in our specimens). It is possible that these differences result from different preservation states of the individual specimens, or that Sauvage [35] believed that he had seen spines on the preopercle and opercle because it was an argument in favour of an attribution to the cottids.

### Phylogenetic Position of †*Lepidocottus* within the Perciformes

The here presented description of the cranial and postcranial skeleton, otolith, and scales of †*Lepidocottus aries*, type species of †*Lepidocottus*, demonstrates that several of the characters are present that define the Gobioidei ([6], [11], [50], [51], see also [56]). They include:

- Hypurals one and two fused, hypurals three and four fused, terminal centrum fused with hypurals three and four;
- Proximal region of the parhypural reduced, so that the bone is separated by a distinct gap from the terminal centrum;
- Lack of parietals;
- Lack of a lateral line on the body;
- Otoliths with a shoe-sole-like sulcus (see [29]).

Thus, †*Lepidocottus* clearly belongs to the Gobioidei, and we can use our new data for the rare opportunity to incorporate a variety of characters from an exceptionally preserved fossil to scrutinize its phylogenetic position among an extant group of fishes.

### Phylogenetic Position of †*Lepidocottus* within the Gobioidei

There is no consensus of the phylogenetic relations and number of families within the Gobioidei (see [19], [58], [59]). Several family-based clades have been introduced, i.e. the Rhyacichthyidae (see [60]), Odontobutidae (see [61]), Butidae and Eleotridae (see [13], [21]), Gobionellidae (Gobionellinae sensu Pezold (1993), see [23]), Gobiidae (see [62]), Milyeringidae (see [18]), and Thalasseleotrididae (see [19]). The Butidae and Eleotridae *sensu* Thacker [13] have been previously treated as subfamilies (Butinae, Eleotrinae) of the Eleotridae (e.g. [1], [5], [7], [63]). Thacker’s families consisted of the same genera as the subfamilies. The Milyeringidae represent small blind cave fishes that were previously assigned to the Butidae and Odontobutidae, respectively [13], [14]. The Thalasseleotrididae comprise two marine genera from Australia and New Zealand that were previously considered as members of the Eleotridae [19].

According to our results, †*Lepidocottus* cannot be assigned to the Rhyacichthyidae because the type species of this family, *Rhyacichthys aspro* (Valenciennes), is clearly different from †*L. aries*. According to Hoese [6] and Hoese and Gill [7], *R. aspro* possesses a lateral line on the body (absent in †*L. aries*), three epurals (two in †*L. aries*), and scales with several rows of transforming ( = distally truncated) ctenii (one row of ctenii and no transforming ctenii in †*L. aries*). The otolith morphology of *R. aspro* is unknown.

Affinities of †*Lepidocottus* with the Odontobutidae are also unlikely, in particular when considering the characters of the scales and otoliths. The Odontobutidae possess one or more rows of transforming ctenii [7], whereas †*L. aries* lacks transforming ctenii (Fig. 3B4). The otolith of the type species of the Odontobutidae, *Odontobutis obscura* (Temminck & Schlegel), is irregular in shape and has a drop-like projection of the ventral rim (Fig. 6E), while the otolith shape is rectangular in †*L. aries* (Fig. 6A). A further member of the Odontobutidae according to Hoese and Gill [7] is *Micropercops swinhonis* (Günther). The otolith of this species clearly differs from the otolith of †*L. aries* because of its triangular shape (Fig. 6F; see also [64]). The differences in otolith morphology between *O. obscura* and *M. swinhonis* may add support to the hypothesis that the Odontobutidae does not represent a monophylum, as suggested in Hoese and Gill [7] and Ahnelt and Göschl [3]. Another possibility is, that not all species currently considered as members of *Micropercops* (of which the type species is *M. dabryi* Fowler & Bean) belong to this genus, as suggested in Iwata [61].

Moreover, †*Lepidocottus aries* cannot be assigned to the Gobiidae and Gobionellidae, despite the fact that it shares with these families the presence of an interneural gap between the two dorsal fins. Gobiidae and Gobionellidae usually have five branchiostegal rays (six in †*L. aries*), a caudal peduncle length that is shorter than the base of the second dorsal fin (longer in †*L. aries*), a T-shaped palatine (L-shaped in †*L. aries*), and no entopterygoid (present in †*L. aries*) [6], [7], [47], [48]. The separation of †*L. aries* from the Gobiidae is also supported by the otoliths because otoliths of Gobiidae (Fig. 6B) usually have a centered sulcus of more limited expansion than that of *L. aries*.

†*L. aries* may thus belong either to the Butidae or Eleotridae, with which it shares the presence of an entopterygoid and an L-shaped palatine. The presence of an interneural gap between the two dorsal fins does not conflict this assignment. Even though the interneural gap is a typical character of the Gobiidae, it also occurs in a few Butidae and Eleotridae [5]. In addition, the insertion of the first two pterygiophores of the second dorsal fin in the same interneural space, as observed in †*L. aries*, is consistent with those few Butidae and Eleotridae that display an interneural gap, but differs from almost all Gobiidae with separate dorsal fins (see [5]).

### Validity of the Genus †*Lepidocottus*

The extant Butidae consist of 11 genera living in freshwater and estuarine habitats of the tropical Indo- and W Pacific, Africa, Asia and Oceania [7], [55] (Table 2). All these genera display at least one important morphological feature that discriminates them from †*Lepidocottus* (Table 2). The extant genera of the Eleotridae of which otoliths are known possess otoliths with a centered sulcus and thus cannot be identical with †*Lepidocottus*. As a result, †*Lepidocottus* is not a synonym of any extant butid or eleotrid and represents a valid genus name.

**Table 2:** Overview of the extant genera of the Butidae with number of species, zoogeographic distribution and habitats (after [7], [49], [52], [55]) and selected morphological differences that separate them from †*Lepidocottus*.

Genus and number of species	Standard length (in cm)	Zoogeography	Habitat	Selected differences vs. †*Lepidocottus*
*Bostrychus* Lacepède (9)	7.5–22	Indo- & West Pacific, Asia, East Atlantic	Estuarine	Scales cycloid, 90 or more in longitudinal row
*Butis* Bleeker (6)	8–14	Indo- & West Pacific, Asia	Marine to estuarine	Lower jaw longer than upper jaw, one epural
*Incara* Rao (1)	<6	India	Estuarine	Pterygiophore formula 3-II II I I
*Kribia* Herre (4)	<3	East Atlantic	Freshwater	One epural
*Ophiocara* Gill (2)	20–27	Indo- & West Pacific, Madagascar	Estuarine	33–42 longitudinal scales
*Oxyeleotris* Bleeker (16)	4–65	West Pacific, Asia, Oceania	Estuarine, freshwater	>60 longitudinal scales
*Odonteleotris* Gill (3)	Up to 28	West Pacific, Asia	Estuarine, freshwater	90 or more longitudinal scales
*Parviparma* Herre (1)	n.a.	Philippines	Freshwater	Pterygiophore formula 4-III II I I
*Pogoneleotris* Bleeker (1)	n.a.	Malaysia	Estuarine	Scales ctenoid plus cycloid body scales, eyes reduced,
*Prionobutis* Bleeker (2)	5–12	West Borneo, Papua New Guinea	Estuarine, freshwater	Pterygiophore formula 3-II II I I
*Typhleotris* Petit (2)	<3	Madagascar	In caves	Blind, small size

Note that *Odonteleotris*, *Parviparma* and *Pogoneleotris* are not listed among the valid genera of Butidae in [21]; their systematic affinities are in need of further investigation. n.a. = data not available.

### †*Lepidocottus*: a Member of the Butidae or Eleotridae? – Comparison of Characters

#### Osteology

The separation of the Butidae and Eleotridae is supported by molecular data [13], but is less clear if anatomical data is taken into consideration [5], [7], [12]. Specializations both of the jaw musculature and caudal cartilage are the known synapomorphies used to characterize the Eleotridae [7]. However, muscles and cartilages are not preserved in fossil gobioids. Moreover, no synapomorphy is presently available to classify the Butidae; rather, this family is currently identified as lacking the apomorphies that define the Eleotridae [7].

The caudal endoskeleton, usually a significant source of diagnostic characters in fish taxonomy, may display apomorphies that help in correctly identifying butid or eleotrid genera [7], [51], [65], but it does not provide any diagnostic feature that can be interpreted as a synapomorphy at the family level [7]. The presence of two epurals and one parhypural, as in †*Lepidocottus aries*, occurs in several butid and eleotrid taxa [7]. The number of branched and segmented caudal rays is 15 in Butidae, with the exception of *Kribia*, which has 11–13 branched and segmented caudal rays [7]. Eleotridae usually possess 13 branched and segmented caudal rays (precisely as in *Kribia* and †*L. aries*).

Characters of the cranium of †*Lepidocottus aries* are the presence of an infraorbital bone (Fig. 4B) and irregular ridges along the orbital margins of the frontal bones (Fig. 4C1). Considering the Butidae and Eleotridae, the only taxa with which †*L. aries* shares an infraorbital bone are *Bostrychus* and *Oxyeleotris* (both belong to the Butidae), and the only taxon with which †*L. aries* shares the irregular ridges on the frontals is *Butis* (data from [52]). It is possible that these two characters represent a synapomorphy of the family Butidae (vs. Eleotridae) because both characters are absent in the Eleotridae (at least what can be said based on the available data). If this is correct, then the presence of these characters in †*L. aries* is indicative of affinities with the Butidae, and the absence of these features in certain butids represents a secondary loss. However, a more complete data set on these characters in extant Eleotridae is necessary to rule out the possibility that the infraorbital bone and irregular ridges along the orbital margins of the frontals represent homoplasies.

An additional interesting character is the **pterygiophore** **formula** of the first dorsal fin; it is 4(2211) in †*Lepidocottus aries*. Both Butidae and Eleotridae include genera in which the first pterygiophore inserts behind vertebra 4 (see [7] and here Table 3), like in †*L. aries*, and thus the position of the first pterygiophore is not significant at family level. However, the **pterygiophore** **arrangement** (2211), as seen in †*L. aries*, is equal to that of most Butidae, with a few exceptions in *Kribia* and *Parviparma* (see [7] and here Table 3). In contrast, *Eleotris* and several other Eleotridae display a (1221) pterygiophore arrangement (see [7], [9] and here Table 3). We agree with previous authors in that the phylogenetic significance of the pterygiophore formula is difficult to grasp, however, the (2211) arrangement of the pterygiophores appears more common in the Butidae than in the Eleotridae and hints to an assignment of †*L. aries* to the Butidae.

**Table 3:** Meristic characters of †*Lepidocottus aries* (this study) and selected extant butid and eleotrid species.

		Extant Butidae						Extant Eleotridae					
	†*Lepidocottus* *aries* *(*Agassiz*)*	*Kribia* *kribensis*(Boulenger)	*Bostrychus* *africanus* (Steindachner)	*Bostrychus* *sinensis* Lacepède	*Butis* *amboinensis*(Bleeker)	*Ophiocara porocephala*(Valenciennes)	*Oxyeleotris marmorata*(Bleeker)	*Eleotris* *acanthopoma* Bleeker	*Eleotris* *fusca* (Forster)	*Eleotris* *melanosoma* Bleeker	*Eleotris* *oxycephala* Temminck & Schlegel	*Giuris* *margaritacea*(Valenciennes)and*Ophieleotris*sp. in [48]	*Hypseleotris* *cyprinoides*(Valenciennes)
**First dorsal fin**	VI	VI (VII)	VI	VI	VI	VI	VI	VI	VI	VI	VI	VI	VI
**Second dorsal fin**	I, 9–10	I, 7–10	I, 9	I, 9–10	I, 8	I, 8	I, 9	I, 8	I, 8	I, 8	I, 8	I, 8	I, 8
**Anal fin**	I, 9	I, 6–9	I, 8	I, 9	I, 9	I, 7	I, 8	I, 8	I, 8	I, 8	I, 8	I, 9	I, 9
**Pectoral fin**	16	14–16	16	17	17	13	17–19	16	18	17	17	15	13
**Pelvic fin**	6	6	6	6	6	6	6	6	6	6	6	6	6
**Longitudinal scales**	50	32–37	78–86	98	31	37	n.a.	51	65	56	50	31	28
**Transverse scales**	10	11–15	31–40	35	9	16	n.a.	16	18	19	19	11	9
**Predorsal scales**	25	0–15	42–58	58	28	26	60–65	41	50	38	48	17	14
**Type of scales** (predorsal+body)	cycloid+ctenoid	cycloid+ctenoid	cycloid	cycloid	ctenoid	ctenoid	ctenoid	cycloid+ctenoid				ctenoid	ctenoid
**Pterygiophore formula** (first dorsal fin)	4(22110)	3(1221)/3(2211)/3(12210)	3(2211)	3(2211)	3(2211)	3(2211)	3(2211)	3(1221)				3(12210)/4(2310)	3(1221)
**Vertebrae** (abdominal+postabdominal)	10+15–16	11+16/12+15	10+16	11+16	12+14	10+15–16	10+16–17	10+15–16				10+15–16	14+11
**Caudal fin** (number of branched rays)	13	11–13	15	15	15	15	15	13				13	13

Data compiled from [5], [7], [48], [49], [52], [53], n.a., data not available.

#### Meristics

The composition of the unpaired and paired fins, the number of longitudinal and transverse scales, as well as the number of vertebrae are not appropriate to separate between Butidae and Eleotridae. Examples are the counts of I8 for the second dorsal fin, I9 for the anal fin, 9 for the number of transverse scales, and 10+15–16 for the precaudal/caudal vertebrae that all are present both in butid and eleotrid species (see Table 3). Moreover, it appears that the only meristic characters that are diagnostic for an individual genus include the number (or ranges) of predorsal and longitudinal scales (Table 3). Adding support to this suggestion comes from the “keys” for the genera of Butidae and Eleotridae [52], in which the number (or ranges) of these scales is used as a key character in the identification of genera. As a result, the number of predorsal or longitudinal scales represents an autapomorphy for a given genus of the Butidae and Eleotridae, but does not discriminate between the families.

#### Scales

The scales of extant butids and eleotrids usually are ctenoid, but several exceptions exist. For example, *Eleotris* possesses small cycloid scales in the predorsal region, at the pectoral-fin base, at the abdomen, and in one or two rows along the borders of the median fins, whereas large ctenoid scales cover the sides of its trunk [66]. The eleotrid *Philypnodon grandiceps* (Krefft) also shows a combination of both scale types along the body, whereas *Erotelis* has exclusively small cycloid body scales [9], [67]. Among the Butidae, *Pogoneleotris* displays ctenoid plus auxiliary cycloid body scales, *Kribia* produces cycloid predorsal scales, but large and ctenoid body scales (with the exception of a few cycloid scales that may appear on the border of the second dorsal fin), while *Bostrychus* is the only butid genus that is covered exclusively by cycloid scales [52], [53]. As a result, the presence or absence of cycloid or ctenoid scales cannot be used for a general discrimination between Butidae and Eleotridae.

However, Pezold and Cage [66] have shown that the occurrence of many small cycloid body scales (termed as secondary cycloid scales in the following) can be interpreted as a derived character in *Erotelis*, while the appearance of large ctenoid scales is regarded as conservative. Accordingly we hypothesize that, during evolution, secondary cycloid scales successively replaced ctenoid scales in both Butidae and Eleotridae. If this hypothesis is accurate, then the many small cycloid scales of *Bostrychus* would be more derived than the scale pattern of any other butid genus. This is consistent with the position of *Bostrychus* on the molecular tree presented by Thacker [13]. As a result, the presence of secondary cycloid scales may represent a tool for determining relationships within the Butidae and Eleotridae.

#### Otoliths

The morphological characters of the saccular otolith (termed otolith in the following) hitherto have received little attention in phylogenetic studies. Otolith formation involves hormonally-regulated calcium carbonate deposition within an organic framework, but otoliths are not part of the skeleton and have evolved independently [68]. Their significance in phylogenetic analyses remains to be fully explored; however, it is long since known that otolith morphology can be characteristic not only at the species level, but also at genus and family level ([29], [69] and many others).

**Figure 7:**
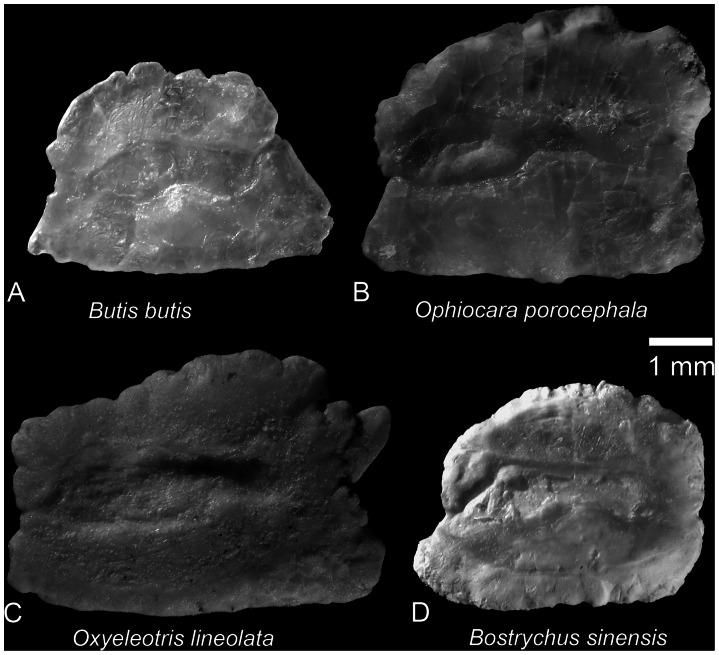
Otoliths of selected extant Butidae (refigured from [65]). **A.** **** *Butis butis* (Hamilton), Sumatra, coll. Schwarzhans, leg. ZMH. **B.** *Ophiocara porocephala* (Valenciennes), Manus Island, Bismarck Archipel, ZMUC P.781771–78. **C.** *Oxyeleotris lineolata* (Steindachner), northern Australia, coll. Schwarzhans, leg. WAM. **D.** *Bostrychus sinensis* Lacepède, China, coll. Schwarzhans, leg. ZMH.

We have compared the otoliths of †*Lepidocottus aries* with the otoliths of those extant butids and eleotrids of which data on otolith morphology are available (Figs. 6, 7). Clearly, the rectangular otolith shape and the anteriorly extended sulcus (with the ostium tip very close to the anterior otolith margin) of †*L. aries* are also present in otoliths of several Butidae (Figs. 6B, 7; see also [30]: Pl. 4, [65]: Figs. 11, 12). In contrast, otoliths of Eleotridae do not show these characters; they are usually quadratic or higher than long (exceptions occur in *Eleotris*) and their sulcus consistently is centered (Fig. 6C, see also [65]).

Among the Butidae, the otolith of †*L. aries* shares the rectangular shape and the anteriorly extended sulcus with *Bostrychus africanus* (Steindachner), *B. strigogenys* Nichols, *B. sinensis* Lacepède (Fig. 7D), *Kribia kribensis* (Boulenger) (Fig. 6B), *Ophiocara porocephala* (Valenciennes) (Fig. 7B), and *Oxyeleotris lineolata* (Steindachner) (Fig. 7C). The otolith of *Butis butis* (Hamilton) is different from the former taxa in that it is rectangular to trapezoid in shape and possesses an almost centered sulcus (Fig. 7A). Otoliths of *Oxyeleotris* species other than *O. lineolata* are rectangular in shape, but their sulcus is centered (figured in [65]: Figs. 12L–N), which might suggest that their assignment to the Butidae may deserve further investigation.

**Figure 8:**
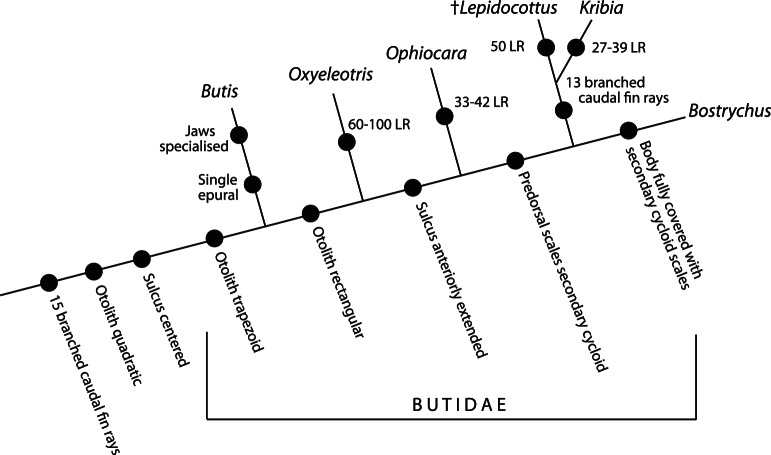
Hypothetical phylogenetic scenario of †*Lepidocottus* within the Butidae. For genus apomorphies we used the number of scales in the longitudinal row (LR) according to Larson and Murdy [52].

The differences between the otoliths of the Eleotridae, and those of the Butidae and †*Lepidocottus aries* raise the question as to whether the characters otolith shape (rectangular in most Butidae and †*L. aries* vs. quadratic or higher than long in most Eleotridae) and position of sulcus (anteriorly extended in most Butidae and †*L. aries* vs. centered in Eleotridae) have phylogenetic implications. The different otolith shapes cannot be explained in terms of function, but "it is possible to argue from parsimony and suggest that the complex shapes are biologically meaningful” ([70]: 502). The biological meaning of a sulcus that is anteriorly extended is also difficult to interpret because the relationship between otoliths and inner ear function in teleost fishes are incompletely understood to date [70]–[73]. Perhaps otoliths with an anteriorly extended sulcus (as seen in †*L. aries* and the butids *Bostrychus*, *Kribia*, *Ophiocara, Oxyeleotris lineolata*) have the advantage of a broader range of sensitivity (such as improved hearing) in comparison to otoliths with a centered sulcus. If this is correct, then the anteriorly extended sulcus represents an apomorphic character for most butid lineages and †*L. aries*. This hypothesis is supported by a study on the oldest record of gobioid otoliths from the Lower Eocene of India [27]. In this paper, two new otolith-based fish species are described and interpreted as belonging to the Gobiidae. One of these is characterized by triangular otoliths, in which the ostium is wider than the cauda (see [27], Fig. 2a–f). These otoliths resemble those seen in present-day Odontobutidae (here Figs. 6E–F), and thus may represent an ancient member of this family, rather than a species of the Gobiidae. The otoliths of the second species, however, are nearly quadratic and have a centered sulcus (see [27], Fig. 2g–o). They resemble the otoliths of certain extant eleotrid and gobiid species, but clearly differ from the otoliths of extant Butidae and †*L. aries*. As a result, the otolith-based fossil record of Gobioidei from the Lower Eocene of India adds support to the hypothesis that the “quadratic shape” and “centered sulcus” are plesiomorphic otolith characters in an ancient lineage of gobioid fishes. Accordingly, the “anteriorly extended sulcus” of several butids and †*L. aries* would represent an apomorphic character. We therefore conclude that †*L. aries* most likely belongs to the Butidae (Fig. 8).

On the other hand, the most recent molecular phylogenetic hypothesis [13] interprets the Eleotridae as the sister of a clade containing the Butidae as sister of the Gobiidae and Gobionellidae. This raises the question as to whether †*L. aries* may represent a stem taxon to the [Eleotridae+[Butidae+[Gobiidae+Gobionellidae]]]. However, the fossil record does not provide support for this hypothesis. Fossil otoliths indicate that the Gobiidae were present in the Middle Eocene of India [74]. The oldest record of Gobiidae from Europe consists of a species of *Pomatoschistus* Gill from the Lower Oligocene of the southern Upper Rhinegraben [75]. Additional evidence of *Pomatoschistus* in the Oligocene of Europe comes from isolated otoliths from the Western Paratethys [76]–[77]. As a consequence, the split leading to the Gobiidae had probably occurred already in the Eocene, and the Oligocene-Early Miocene taxon †*Lepidocottus* cannot be regarded as a stem taxon to the [Eleotridae+[Butidae+[Gobiidae+Gobionellidae]]]. Based on the otolith data, osteological characters and the fossil record of the Gobioidei, we conclude that †*Lepidocottus* is closely related to the extant Butidae and represents the first skeleton-based record of this group.

### †*Lepidocottus*: Hypothetical Position within the Butidae

We hypothesize a phylogenetic setting that uses the presence of 15 branched caudal fin rays, quadratic otoliths and a centered sulcus as plesiomorphic characters (Fig. 8). The differentiation of the otolith shape is considered a synapomorphy for the Butidae. The trapezoid otolith shape is defining the *Butis* lineage, while rectangular otoliths are interpreted as a synapomorphy for a clade that comprises †*Lepidocottus* and the extant Butidae, with the exception of *Butis*. The differentiation of the sulcus, i.e. appearance of an anteriorly extended sulcus, is a synapomorphy for †*Lepidocottus* and the extant Butidae, except *Butis* and *Oxyeleotris*. Secondary cycloid predorsal scales characterize a clade consisting of †*Lepidocottus, Kribia* and *Bostrychus*, with *Ophiocara* as sister. In addition, †*Lepidocottus* is interpreted as sister to *Kribia* based on the reduced number of caudal fin rays (13 vs. 15 in other butids).

Our phylogenetic hypothesis suggests an early divergence of *Butis* from the other butids, which is consistent with the molecular phylogeny of Thacker [13]. In addition, *Oxyeleotris* appears to diverge earlier than *Ophiocara*, which also does not conflict the molecular phylogeny of Thacker [13]. A difference between our scenario and Thacker’s phylogeny concerns the position of *Bostrychus* as sister to *Kribia* (*Bostrychus* is sister to *Ophiocara* and *Kribia* is positioned within a polyphyletic *Oxyeleotris* clade in Thacker).

### The Fossil Record of †*Lepidocottus*

Two further †*Lepidocottus* species have been described previously based on articulated skeletons:

- †*Lepidocottus papyraceus* (Agassiz) from the Lower Oligocene of Italy;
- †*Lepidocottus gracilis* Laube from freshwater sediments of the Lower Oligocene of eastern Germany [78], [79].

In addition, three otolith-based species, in previous studies described as “*Gobius*” and/or “genus Eleotridarum” (in the old definition, i.e. including the present-day Eleotridae and Butidae) can now be identified as belonging to †*Lepidocottus*:

- †*Lepidocottus martinii* (Reichenbacher & Uhlig) from Upper Oligocene brackish deposits in the western Paratethys (see [77], as “genus Eleotridarum”);
- †*Lepidocottus schadi* (Weiler) from Upper Oligocene brackish deposits of the southern Upper Rhinegraben (see [80], [81], as *Gobius* and “genus Eleotridarum”);
- †*Lepidocottus sectus* (Stinton & Kissling) from Upper Oligocene and Lower Miocene brackish and freshwater deposits in the western Paratethys (see [82], as “genus Eleotridarum”).

A single marine †*Lepidocottus* species has been described from the coastal Lower Miocene (Aquitanian) site La Paillade in southern France (see [31], as “genus Eleotridarum” *sectus*]. This species probably represents †*L. martinii*, rather than †*L. sectus*. This record represents the only known case in which †*Lepidocottus* co-occurs with numerous Gobiidae [31]. Evidence of †*Lepidocottus* younger than Early Miocene (Aquitanian) remains unknown to date.

### Conclusions

Sauvage [35] assigned †*Lepidocottus* to the Cottidae, whereas Gaudant [36], [37] placed the taxon in the Gobiidae and the genus *Gobius* Linnaeus, and consequently regarded †*Lepidocottus* as a junior synonym of *Gobius*. We have shown that †*L. aries* most likely belongs to the Butidae and that several †*Lepidocottus* species thrived in Europe during the Oligocene and Early Miocene (Aquitanian). Thus, a gobioid family that is today largely restricted to W-Africa, the Indo- and West-Pacific and not present in Europe or in the Mediterranean Sea was a common member of the fossil fish faunas during the Oligocene and Early Miocene in Europe (Mediterranean area, Paratethys, Upper Rhine Graben). Similar to the recent Butidae, these fossil fishes were adapted to a wide range of salinities and thrived in freshwater, brackish and, more rarely, in marginal marine habitats. Their disappearance from Europe and the Mediterranean and Paratethys areas, respectively, probably occurred during the Early Miocene (Aquitanian) and may be linked with the apparent radiation of the Gobiidae during that period of time.
